# Causality Assessment of Adverse Drug Reaction Toxic Epidermal Necrolysis With the Aid of ChatGPT: A Case Report

**DOI:** 10.7759/cureus.60638

**Published:** 2024-05-19

**Authors:** Sajal Pandya, Chetna Patel, Brijesh Sojitra, Hetal Karamata

**Affiliations:** 1 Pharmacology, Government Medical College and New Civil Hospital, Surat, IND; 2 Pharmacology and Therapeutics, Government Medical College and New Civil Hospital, Surat, IND; 3 Pulmonary Medicine, Government Medical College and New Civil Hospital, Surat, IND

**Keywords:** causality assessment, scroten score, chatgpt, pharmacovigilance, toxic epidermal necrolysis

## Abstract

Toxic epidermal necrolysis (TEN) is a severe and potentially fatal adverse drug reaction. This case report presents a 19-year-old male with pulmonary tuberculosis undergoing anti-tubercular therapy who developed TEN. The patient had multiple comorbidities including type 1 diabetes mellitus and multisystem atrophy. ChatGPT was utilized alongside conventional methods to assess causality. While conventional scoring systems estimated mortality at 58.3% (SCORTEN) and 12.3% (ABCD-10), ChatGPT yielded divergent scores. Causality assessment using WHO-Uppsala Monitoring Centre (UMC) and Naranjo's scale indicated rifampicin and isoniazid as probable causative agents. However, ChatGPT provided ambiguous results. The study underscores the potential of AI in pharmacovigilance but emphasizes caution due to discrepancies observed. Collaborative utilization of artificial intelligence (AI) with clinical judgment is advocated to enhance diagnostic accuracy and treatment decisions in adverse drug reactions. This case highlights the importance of integrating AI into drug safety systems while acknowledging its limitations to ensure optimal patient care.

## Introduction

The primary objective of pharmacovigilance is safe and efficient medication utilization in the public health care system. Reporting the adverse drug reaction, causality assessment, and generating a database of safety information on drugs are a part of pharmacovigilance [1].

Previously known as Lyell syndrome which is Stevens-Johnson syndrome (SJS) or toxic epidermal necrolysis (TEN) is a rare, acute, serious, and potentially fatal cutaneous adverse drug reaction in which there is sheet-like skin and mucosal loss accompanied by systemic symptoms of hypersensitivity reactions [2]. SJS/TEN was reported as a cutaneous adverse drug reaction due to antibiotics like sulfa drugs, anti-epileptics including phenytoin, carbamazepine, lamotrigine, phenobarbital, and non-steroidal anti-inflammatory drugs, etc. [3]. 

AI can significantly enhance medication therapy management (MTM) by efficiently identifying drug interactions. Artificial intelligence (AI) systems like ChatGPT can swiftly pinpoint potential interactions and suggest appropriate interventions by integrating patient data with extensive drug databases. This application of AI not only streamlines MTM processes but also leverages the vast amount of medical knowledge available to improve patient care. In simpler terms, AI, like ChatGPT, can help healthcare providers identify and address medication issues faster and more accurately, ultimately leading to better patient outcomes [4]. ChatGPT offers precise and current details on medications, covering aspects like dosages, purposes, potential side effects (ADR), interactions with other drugs, and even cost information. With this comprehensive data, patients, and pharmacists can make well-informed decisions when choosing treatment options. ChatGPT acts as a reliable resource, empowering individuals to navigate their healthcare choices with confidence and clarity [5]. The purpose of this case report is to highlight the rifampicin- and isoniazid-induced adverse drug reaction SJS and causality assessment of this ADR by WHO-Uppsala Monitoring Centre (UMC) scale, Naranjo's scale, analysis of the severity of SJS by Score of TEN (SCORTEN), ABCD-10 (age, bicarbonate, cancer, dialysis, 10% body surface area) score. Analysis was also done by ChatGPT which was compared with other results of the analysis.

## Case presentation

A 19-year-old male patient microbiologically confirmed case of pulmonary tuberculosis came to the emergency department with complaints of high-grade fever, erosions of the oral mucosa, peeling of skin over the dorsum of hands and foot for three days, extremely severe pain for one day admitted to the intensive care unit of the respiratory medicine department. A patient who has been on first-line anti-tubercular therapy for one month developed generalized macules over the body, peeling of skin over the dorsum of hands and foot, and oral ulcers dermatologists diagnosed as TEN. The patient's medication information has been documented (Table 1).

**Table 1:** Drug details of the patient. RBS: Random blood sugar, OD: Once a day.

Sr. No.	Drug name	Dose	Frequency	Formulation	Duration	Route of administration	Indication
Suspected drug							
1	Isoniazid	150 mg	OD	Tablet	6 months	Oral	Pulmonary tuberculosis
2	Rifampicin	300 mg	OD	Tablet	6 months	Oral	Pulmonary tuberculosis
Concomitant drug							
1	Pyrazinamide	600 mg	OD	Tablet	2 months	Oral	Pulmonary tuberculosis
2	Ethambutol	450 mg	OD	Tablet	6 months	Oral	Pulmonary tuberculosis
3	Rivaroxaban	10 mg	OD	Tablet	6 months	Oral	Partial pulmonary thrombosis
4	Plain insulin	According to RBS		Injection	Lifelong	Subcutaneous	Type-1 diabetes mellitus

A patient has a history of type-1 diabetes mellitus, ectopic malrotation of the kidney, urethral stricture before six months, multisystem atrophy-cerebellar type (MSA-C), cervical spine and L4-L5 disc prolapse without neural involvement. Partial pulmonary embolism and microbiologically confirmed case of pulmonary tuberculosis. Family history was not significant.

**Figure 1:**
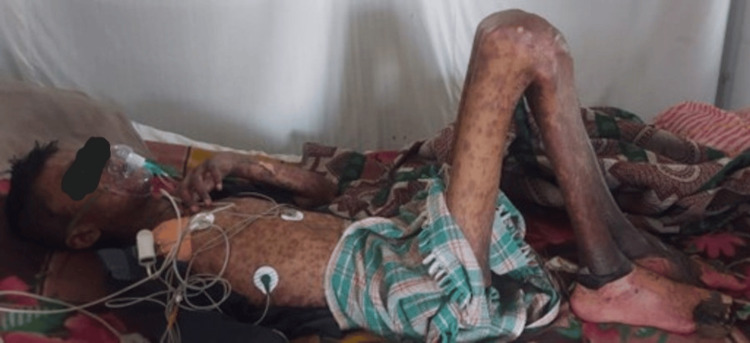
Generalized multiple hyperpigmented macules present over the chest, abdomen, back, thighs, legs, bilateral arms and forearms, and genitals. Few bullae are present over the right hand. Painful bleeding spots are present. Exfoliation of skin over the dorsum of hands and feet.

**Figure 2:**
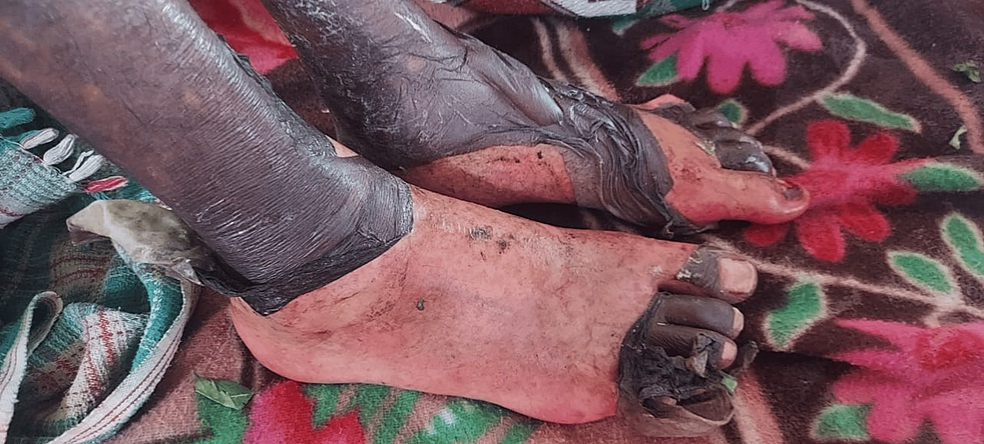
Bullae and exfoliation of skin over the dorsum of the bilateral feet.

The patient was conscious, cooperative, and well-oriented to time, place, and person. Generalized multiple hyperpigmented macules were present over the chest, abdomen, back, thighs, legs, bilateral arms and forearms, and genitals. Few bullae were present over the right hand. Painful bleeding spots were also present. There was exfoliation of skin over the dorsum of hands and feet (Figures 1, 2). 

On fundus examination the total pallor of the disc and media were clear, blood vessels were normal around the disc and maculo-foveolar reflex was seen. On oral examination, poor oral hygiene, angular cheilitis, and stomatitis were seen and the oropharynx was congested.

The probable diagnosis was septic arthritis while the final diagnosis was acute respiratory failure with septicemia with septic shock and an immediate need to assess a cause of TEN. Microbiologically confirmed case of pulmonary tuberculosis with a known case of type 1 diabetes mellitus, MSA-C, cervical spine, and L4-L5 disc prolapse without neural involvement and partial pulmonary thrombosis.

Insertion of a central venous catheter (CVP) and catheterization were done at the time of admission. The patient was advised to have an anti-diabetic diet, a high-protein diet, and concentrated ORS (oral rehydration solution). Hepatotoxic, oculotoxic, and nephrotoxic drugs and glucose water were avoided. Proper antibiotic coverage along with electrolyte balance were given. The patient was on non-invasive ventilatory (NIV) support after admission worsening the condition due to septicemia patient’s relative had been given negative consent for intubation. The basic laboratory investigations for this patient were done (Table 2).

**Table 2:** Laboratory investigation of the patient. NR: Non-reactive.

Test	Patient’s result	Reference range	Unit
Hemoglobin	7.6	14.0-17	gm/dl
Total count	14600	4000-11000	cumm
Platelet count	454000	150000-450000	cumm
Peripheral smear malaria parasite	Negative		
Random blood sugar	304	70-110	mg/dl
Albumin	2	3.5 to 5.5	gm/dl
Bilirubin			
Direct	0	0.0 to 0.3	mg/dl
Indirect	0.2	0.2 to 0.8	mg/dl
Total	0.2	0.3 to 1.0	mg/dl
Serum creatinine	0.99	0.74 to 1.35	mg/dl
Serum potassium	4.72	3.5 to 5.5	mEq/liter
Serum sodium	125.7	135 and 145	mEq/liter
Total protein	4.5	6-8	g/dl
Erythrocyte sedimentation rate	84	1-7	mm/hour
Prothrombin time	15.9	10-13	seconds
International normalized ratio	1.15	1.1 or below	
Serum urea nitrogen	25 mg/dl	8-23	mg/dl
Serum bicarbonate	18 mmol/L	22-29	mEq/liter
D-dimer	1161	<0.50	mg/dl
Amylase	27	40-140	U/liter
Lipase	3	10-140	U/liter
HIV	NR		
Sputum acid fast bacilli	Positive		

For the management of skin lesions withdrawal of the suspected drugs rifampicin and isoniazid, cleaning of the lesion with normal saline, local application of the fusidic acid cream, and application of the vaseline gauze. Continuing follow-up with the treatment may improve the skin lesions newer skin lesions were not developed after the admission. Re-introduction of the suspected drugs was not done.

The patient presented with bilateral extensive pulmonary tuberculosis evident on chest X-ray. Ultrasonography revealed a fusion of the right kidney and visualization of the left kidney in the lung region, indicative of ectopic malrotation of the kidney. A high-resolution computed tomography (CT) scan of the thorax showed non-enhancing hypodense material in the right lower lobe with a probability of pulmonary artery thrombosis. Additionally, a wedge infarction was observed in the right lower lobe. The etiology of the acute infection was likely Koch's infection.

## Discussion

SCORTEN is a widely recognized tool specifically designed to assess the severity of SJS and toxic TEN cases. It consists of seven clinical and biological parameters, such as age, presence of malignancy, and extent of skin detachment, which are used to predict the likelihood of mortality during hospitalization. SCORTEN's predictive power ranges from a 3.2% to a high 90.0% probability of mortality, making it a valuable tool for clinicians to gauge the severity of the condition. Beyond its main purpose of predicting patient outcomes, SCORTEN also serves other important functions. It's commonly used as a reference point in clinical trials to evaluate the effectiveness of different treatments aimed at modulating the immune response. Additionally, SCORTEN serves as a benchmark for assessing the quality of care provided across different healthcare centers worldwide. This multi-faceted role underscores the significance of SCORTEN in guiding treatment decisions and assessing the overall management of SJS and TEN cases. ABCD-10 score is the prognostic tool that has been developed and tested in the United States to predict mortality in cases of epidermal necrolysis. This score takes into account factors such as the patient's age, bicarbonate levels, history of cancer, need for dialysis, and the extent of skin involvement (measured as a percentage of the body surface area). By considering these factors, the ABCD-10 score aims to provide healthcare professionals with valuable insights into the likelihood of mortality in patients with epidermal necrolysis [6].

SCORTEN score for this patient was 4 and the ABCD-10 score was 2 which showed estimated mortality for this patient was 58.3% and 12.3% respectively. The analysis with the aid of ChatGPT indicated SCORTEN score for this patient was 498, and the ABCD-10 score was approximately 39.04. Based on that estimated mortality rate was not calculated, it was described as an extremely high SCORTEN score of 498, so the mortality risk for this patient would likely be very high. It's important to note that these scoring systems are used as aids in clinical decision-making and prognosis estimation, and other factors, such as the patient's overall health status, comorbidities, and response to treatment, also play crucial roles in determining outcomes. Therefore, prognosis and mortality risk should be discussed with the patient's healthcare team who have access to the full clinical picture. For ABCD-10, the interpretation may vary, but generally, a higher score indicates increased severity and potentially increased mortality risk. However, specific mortality risk estimates based solely on the ABCD-10 score might not be as established compared to SCORTEN [7]. Causality assessment essentially involves determining whether there's a cause-and-effect relationship between a drug and a drug reaction. It's an evaluation to gauge how likely it is that a specific treatment caused an observed adverse event (AE) [8]. Causality assessment by the WHO-UMC scale shows there is a temporal relationship between rifampicin and isoniazid with TEN. Administration of rifampicin and isoniazid seemed to be causally related to the development of life-threatening SJS/TEN. With drug withdrawal, de-challenge results were positive too. This pointed towards the fact that rifampicin and isoniazid may be the suspected drugs for this adverse drug reaction. However, a rechallenge was not performed as it would not be ethical to subject the patient once again to such life-threatening adverse drug reactions. Desensitization refers to a therapeutic process used in medicine to reduce or eliminate allergic or adverse reactions to specific substances, such as drugs, foods, or environmental allergens. So, rifampicin and isoniazid were probable causes of severe cutaneous adverse drug reaction toxic epidermal necrolysis in this patient [9]. Based on the WHO-UMC scale, ChatGPT result was “there is a strong likelihood of a causal relationship between the administration of rifampicin and isoniazid and the development of toxic epidermal necrolysis.” There is no clear-cut analysis and relation shown by the ChatGPT. According to Naranjo’s scale rifampicin and isoniazid were probable causes of TEN, as the score was 7 [10]. Based on Naranjo's algorithm, there is a probable causal relationship between the administration of rifampicin and isoniazid and the development of toxic epidermal necrolysis with ChatGPT. In both assessments, it appears likely that rifampicin and isoniazid were the causative agents for the toxic epidermal necrolysis in this patient. However, it's important to consider these assessments in conjunction with clinical judgment and other available evidence which ChatGPT suggests.

Using AI like ChatGPT to assess if a drug caused a reaction like TEN could improve how accurately we diagnose it and decide on treatment. However, we need to think carefully about how AI fits into our drug safety systems to make sure patients stay safe and get the best care possible [5].

However, it's essential to acknowledge the limitations of ChatGPT in our analysis. The significant deviation in SCORTEN and ABCD-10 scores suggests potential inaccuracies, highlighting the need for caution when relying solely on AI for complex medical assessments, especially in critical scenarios like TEN [11].

## Conclusions

While artificial intelligence (AI) like ChatGPT shows promise in assessing adverse drug reactions such as toxic epidermal necrolysis, its limitations must be recognized. The divergence in scores compared to conventional methods like the WHO-UMC scale, Naranjo's scale, SCORTEN score, and ABCD-10 score indicates potential inaccuracies. Therefore, caution is warranted when employing AI for complex medical assessments. Collaborative utilization of AI alongside clinical judgment and established protocols can enhance diagnostic accuracy and treatment decisions, ultimately improving patient outcomes in pharmacovigilance.
